# Cracking Behavior of René 104 Nickel-Based Superalloy Prepared by Selective Laser Melting Using Different Scanning Strategies

**DOI:** 10.3390/ma13092149

**Published:** 2020-05-06

**Authors:** Kai Peng, Ranxi Duan, Zuming Liu, Xueqian Lv, Quan Li, Fan Zhao, Bing Wei, Bizhong Nong, Shizhong Wei

**Affiliations:** 1 State Key Laboratory of Powder Metallurgy, Central South University, Changsha 410083, China; pengkaicc@163.com (K.P.); RXD742@bham.ac.uk (R.D.); lvxueqian@163.com (X.L.); qli019@sjtu.edu.cn (Q.L.); zhao.fan@cxtc.com (F.Z.); weibing0522@126.com (B.W.); nongbizhong@csu.edu.cn (B.N.); 2 Engineering Research Center of Tribology and Materials Protection, Ministry of Education, Henan University of Science and Technology, Luoyang 71003, China; wsz@haust.edu.cn

**Keywords:** selective laser melting, scanning strategy, René 104 Ni-based superalloy, cracking, residual stress

## Abstract

Eliminating cracks is a big challenge for the selective laser melting (SLM) process of low-weldable Nickel-based superalloy. In this work, three scanning strategies of the snake, stripe partition, and chessboard partition were utilized to prepare René 104 Ni-based superalloy, of which the cracking behavior and the residual stress were investigated. The results showed that the scanning strategies had significant effects on the cracking, residual stress, and relative density of the SLMed René 104 superalloy. The scanning strategies with more partitions boosted the emergence of cracks, as high-density cracks occurred in these samples. The overlapping zone (OZ) of the scanning partition was also susceptible to cracking, which increased the size, number, and density of the cracks. The cracking performance was relatively moderate in the snake-scanned samples, while that in the chessboard-partition-scanned samples was the most severe. It is concluded that the partition scanning strategies induced more cracks in the SLMed René 104 superalloy, of which the residual stress was apparently reduced. Therefore, it is necessary to design scanning strategies with optimized scanning partitions and overlaps to avoid cracking and acquire a high-quality, near fully dense, low-weldable Nickel-based superalloy using SLM.

## 1. Introduction

Selective laser melting (SLM) can fabricate near-net-shape metallic parts by using three-dimensional (3D) computer-aided design data and shows excellent advantages in the preparation of high-performance and complex-shape components [1,2,3,4]. Currently, SLM has been applied to the preparations of various alloys, such as titanium alloys [5,6,7,8], aluminium alloys [9,10], steel [11,12,13], Ni-based alloys [14,15], and high-entropy alloys [16]. However, the significant temperature gradient, fast cooling rate, and repeated re-melting processing, during SLM, lead to excessive-high residual stress in the as-fabricated parts, resulting in warpage and cracks [17,18]. The warpage and cracks degrade the mechanical properties of SLM parts [19,20,21,22], and bring challenges to the SLM fabrication of high-quality components.

Cracking is a typical defect in additive-manufactured alloys, especially in the hard-to-weld superalloy, which leads to severe quality reduction, and even early damage or failure. In terms of the cracking behavior during SLM, the current research focuses on parameter optimization, composition improvement, secondary-phase nanoparticle addition, and post-treatment. Kontis et al. [23] suggested the cracking behavior of non-weldable nickel-based superalloys can be avoided by adjusting the build parameters to obtain a fine-grained microstructure. Pre-heating can also effectively inhibit cracking by reducing the residual stress in laser solid forming IN738LC [24]. Lu et al. [21] reported that chessboard partition scanning effectively reduced residual stress and crack formations of Inconel 718 Ni-based superalloys. In other works, Tomus et al. [25] investigated the effect of minor alloying elements (Si, C and Mn) on crack-formation characteristics of Hastelloy-X. The reduction of these minor alloying elements resulted in the elimination of hot cracks in the laser powder bed fusion (LPBF) fabricated Hastelloy-X, while this modification degraded the tensile strength of about 140 MPa [26]. Increasing the most potent solid solution strengthening elements was also shown to reduce cracks by 65% in SLMed Hastelloy-X alloy [27]. Han et al. [28] revealed that the addition of TiC nanoparticles could eliminate micro-cracks, but 0.14% residual pores were formed in the LPBF-fabricated Hastelloy-X samples. Cracks in SLMed tungsten (W) were also effectively suppressed by the addition of ZrC nanoparticles [29]. However, Qiu et al. [30] proved that cracks in Inconel 738LC, fabricated by SLM, were associated with pores, or Al-, Si-, and W-based oxide particles with high melting point and small grains along some large grain boundaries (GBs). Recently, the crack-free IN738LC superalloy was successfully fabricated by SLM [31], which proved that the high-quality non-weldable Ni-based superalloy could be obtained through systematic optimization of the pre-processing, study of the build parameters, and post-treatment.

René 104 superalloy is the third-generation powder metallurgy (PM) Ni-based superalloy developed by NASA [32] that performs as the optimum structural material for hot-ending components, such as aircraft engine and gas turbine disks, due to the excellent combination of strength, damage tolerance, and durability at elevated temperatures [33]. René 104 superalloy is processed through the PM route, including alloy atomization, hot isostatic pressing, extrusion, and superplastic isothermal forging. However, the traditional PM processing method is restricted to fabricating complex-shaped parts, and new forming technologies must be developed. In recent years, additive manufacturing has become an essential and promising method for solving the problems mentioned above. However, the poor weldability of René 104 alloy made it easy to crack during laser processing. Once the high-density cracks were formed, they were difficult to be eliminated with post-treatment, especially for the parts with complex shapes. Therefore, studying the law of cracking behavior during laser forming for the preparation of considerable size and complex-shaped superalloy components is becoming crucial. Duan et al. [34] investigated the effects of energy density on the cracking behavior of René 104 superalloy and concluded that high residual stresses, caused by excessive energy input, led to the cracks in the samples prepared by SLM. Yang et al. [35] reported that the cracking sensitivity of the René 104 samples fabricated by the laser solid forming (LSF) process closely depends on the heat input, the cracks cannot be eliminated by optimizing the LSF parameters. Controlling the pre-heating and cooling process was helpful in reducing cracks in LSFed René 104 superalloy parts [36], whereas the thermal-induced micro-cracking in certain alloys cannot be fully eliminated by processing optimization [27]. Based on the limited understanding of cracking behavior of as-SLMed René 104 alloy, it is of great importance to explore the influence of scanning strategies on crack elimination during SLM of René 104 alloy to acquire high-quality Ni-based superalloy components.

In this study, based on the SLM process parameter optimization achieved in our early research [34], the three scanning strategies of the snake, stripe partition, and chessboard partition were used in SLM fabrications of René 104 superalloy, of which the cracking behavior and residual stress performance were studied. The results concluded some critical findings for cracking control of René 104 superalloy prepared by SLM.

## 2. Materials and Methods

**Figure 1:**
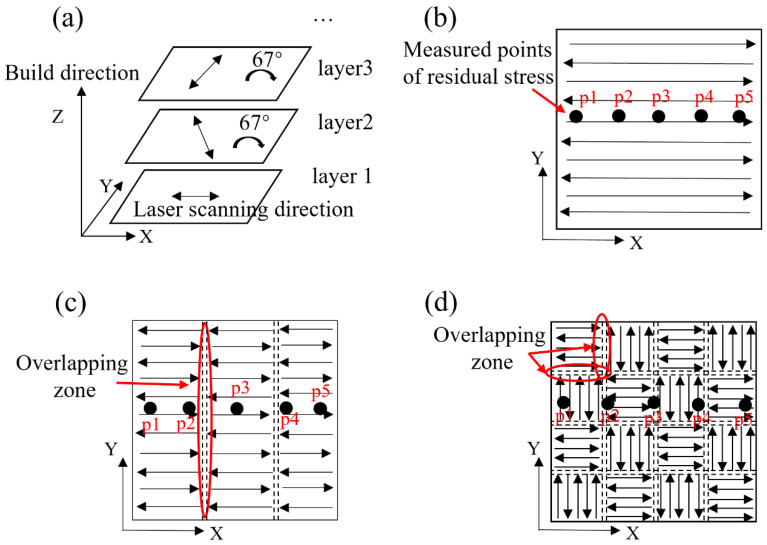
Schematic of the scanning strategies for selective laser melting (SLM) and residual stress measured points (p1, p2, p3, p4, and p5); (**a**) laser scanning route; (**b**) snake scanning; (**c**) stripe partition scanning; and (**d**) chessboard partition scanning.

Snake, stripe partition, and chessboard partition scanning strategies were used to process René 104 Ni-based superalloy powder by SLM using a laser melting facility (FS271M, Hunan Farsoon High-Technology, Changsha, China). Pure argon was used as shielding to ensure that the oxygen content was ≤300 ppm during the laser processing; a heating board was used to heat the substrate to 100 °C; an infrared thermometer was used to monitor the temperature of the substrate in real-time. A schematic of the fabrication process is shown in Figure 1. The building layer was rotated counterclockwise by 67° compared to the previous layer (Figure 1a), and a new layer of powder was dispersed evenly upon the previous build layer. The specimen is a cube plate with dimensions of 20 × 20 × 6 mm. In the build process, the scanning parameters were a laser spot diameter of 120 µm, laser power of 400 W, laser scanning velocity of 800 mm/s, line spacing of 0.12 mm, and layer thickness of 30 µm. The stripe width of the stripe partition scanning strategy (Figure 1c) was 7 mm, and the overlap width between stripes was 0.12 mm. The grid in the chessboard partition scanning strategy (Figure 1d) was 5 × 5 mm, and the overlap width between the grids was 0.08 mm.

**Figure 2:**
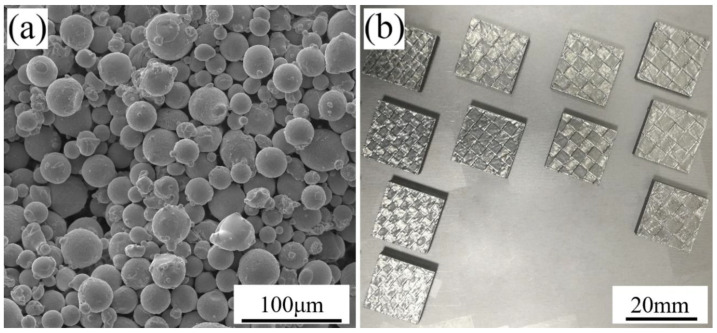
(**a**) Atomized René 104 Ni-based superalloy powder; (**b**) SLMed René 104 Ni-based superalloy samples.

Spherical René 104 superalloy powder, shown in Figure 2a, with a particle size ranging from 15 to 45 µm, an average particle size of 32 µm, an apparent density of 4.08 g/cm^3^, and good flowability was used as the starting material, which was prepared by UK PSI close-coupled gas atomization equipment in our group [37]. The as-SLMed samples are shown in Figure 2b and were cut out from the substrate by wire-electrode cutting. In order to obtain the original cracking and true residual stress, no heat treatment was carried out on the René 104 samples fabricated by different scanning strategies.

The composition of atomized René 104 superalloy powder is shown in Table 1 and was analyzed using a plasma emission spectrometer (IRIS advantage 1000, Thermo Fisher Scientific, MA, USA) and oxygen, carbon, and nitrogen testers (LECO, TC-436, MI, USA).

The crack size, number, density, and distribution of the as-fabricated samples were observed using an Optical Microscopy (OM, DM4500P, Leica Microsystems, Wetzlar, Germany). The OM specimens were prepared by electrolytic corrosion under constant 5 V voltage for 40–100 s, using a water solution of 70% phosphoric acid at room temperature. Residual stress in the X–Y plane of the samples was measured by the PROTO iXRD diffractometer (Proto Manufacturing, Oldcastle, Canada) according to the BS EN15305 standard [38], using the Mn Kα X-ray, Bragg angle of 151.88°, and the wavelength of 2.103 nm. The residual stress test points of p1, p2, p3, p4, and p5 are shown in Figure 1b–d. The distance between each test point was 4 mm, and points p1 and p5 were 2 mm from the edge. The density measurement was performed in accordance with the Archimedean principle of the ISO 3369 standard [39].

## 3. Results and Discussion

### 3.1. Cracks and Their Distribution

**Figure 3:**
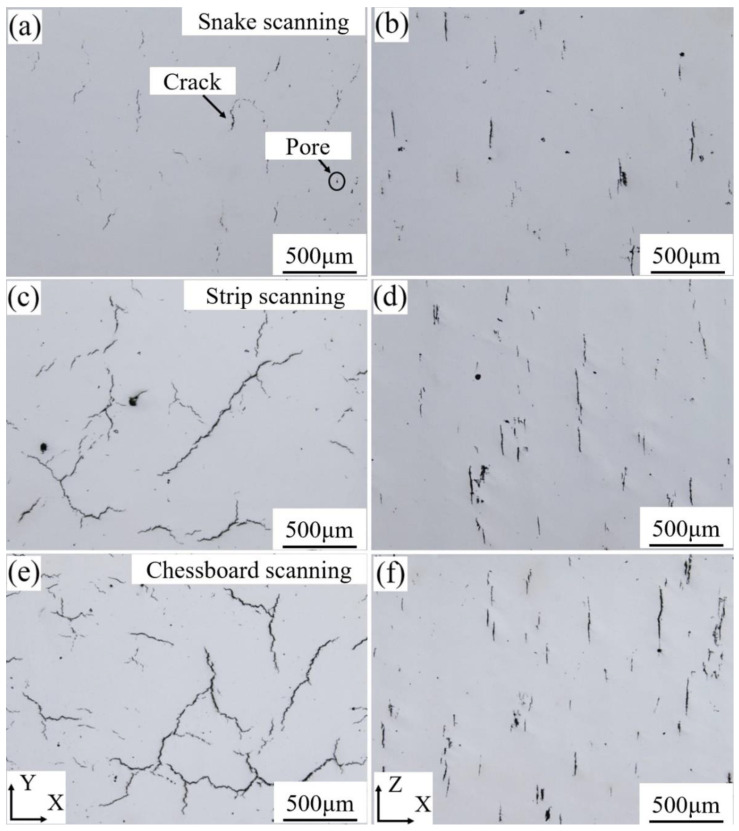
Cracks in the cross-section of the X–Y and X–Z planes of René 104 superalloy samples fabricated by SLM using different scanning strategies; (**a**,**b**) snake scanning; (**c**,**d**) stripe partition scanning; (**e**,**f**) chessboard partition scanning.

Figure 3 shows the observation results of the cracking of SLMed René 104 superalloy samples. The cracking phenomenon occurred on the X–Y and X–Z planes of the samples, fabricated using the three scanning strategies, and a few pore defects were also observed. The cracking degree of the snake-scanned sample was relatively lower (Figure 3a,b), the crack size of the X–Y plane of the as-fabricated sample was mainly concentrated within the range of 20 to 192 µm, and the crack was serrated (Figure 3a). The crack of the X–Z plane was 32–204 µm in length and flat (Figure 3b). The crack size of the X–Y plane of the stripe-partition-scanned sample (Figure 3c) was within the range of 27 to 800 µm, and a few cracks were connected to one other and distributed in a river-like pattern. The crack size of the X–Z plane (Figure 3d) was within the range of 35 to 254 µm, with the number, size, and density of cracks being higher than that of the snake-scanned sample (Figure 3b). The cracking phenomenon of the chessboard-partition-scanned sample (Figure 3e,f) was the most severe, and the number, size, and density of cracks were higher than those of the sample fabricated using snake scanning (Figure 3a,b) and stripe partition scanning (Figure 3c,d). The crack size was up to 1400 µm, within the range of 50 to 1400 µm and the cracks were interconnected and mostly netted on the X–Y plane of the as-fabricated sample (Figure 3e). The crack size of the X–Z plane (Figure 3f) was within 23–461 µm.

The relative densities of the samples fabricated by the snake, stripe partition, and chessboard partition scanning were 98.75%, 98.17%, and 98.01%, respectively. The results are consistent with the cracking result of the samples in Figure 3.

**Figure 4:**
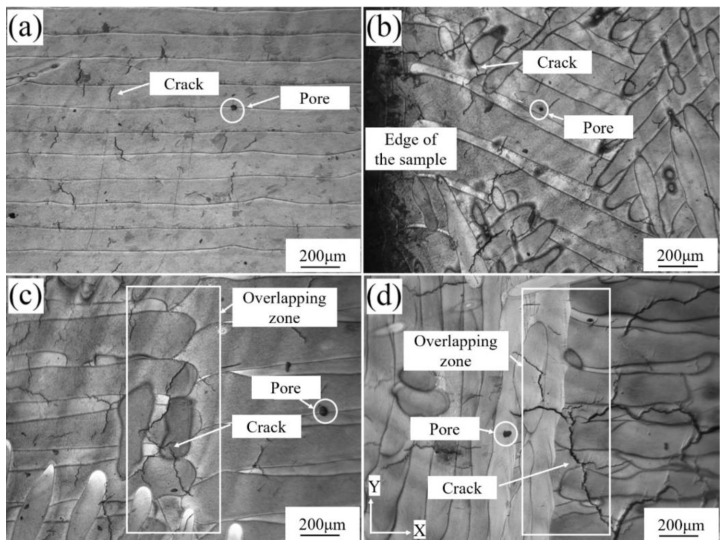
Metallographic microstructure of SLMed René 104 superalloy in the X–Y plane of the samples; (**a**) snake scanning; (**b**) merged molten pool at the edge of the snake scanning; (**c**) overlapping zone of the stripe-partition-scanned molten pool; and (**d**) overlapping zone of the chessboard-partition-scanned molten pool.

In order to further observe the cracking behavior, the metallographic microstructure of René 104 superalloy in the X–Y plane of the as-fabricated sample was investigated, as shown in Figure 4. The cracks were observed in all the as-fabricated samples, and the cracks extended along the normal direction of the molten pool. The merged molten pool at the end of the molten pools cracked more significantly, especially in the overlapping zone (OZ) of the scanning partition. A few pores were also observed in the as-fabricated samples.

The molten pools in the X–Y plane of the sample fabricated by snake scanning showed many cracks with a length of 20–130 µm and a few pores (Figure 4a). The merged molten pool, formed by two adjacent molten pools at the edge of the as-fabricated sample, was more prone to cracking and presented cracks with a length of up to 380 µm, crossing through 1–2 merged molten pools (Figure 4b). The OZ of the scanning partition in the X–Y plane of the sample fabricated by stripe partition scanning, which was a lap joint of two sets of the parallel molten pool end, showed cracks of up to 460 µm (Figure 4c). However, the crack size on the single interior molten pool was small, with a maximum length of 250 µm. The OZ of the scanning partition in the X–Y plane of the sample, fabricated by chessboard partition scanning, which was the connection between the side of one molten pool group and the end of another molten pool group (Figure 4d), showed cracks with a maximum length of 700 µm through 2–4 merged molten pools. With the increase in the number of scanning partition zones, the number of OZs increases, resulting in an increase in the crack number and size. There were a greater number of partition zones in the chessboard partition scanning than in the stripe partition scanning, and the cracking phenomenon of the merged molten pool was the most severe in the chessboard-partition-scanned samples.

The cracking degree of the merged molten pool was significantly different among the samples fabricated by the three scanning strategies and was closely related to the OZ and heat-affected zone (HAZ). SLM is a track-by-track deposition process, which is accompanied by the evolution of residual stresses caused by localized heating and cooling [40], and the stress state of the OZ and HAZ is highly complex due to repeated rapid heating and cooling [41]. This causes the OZ to be susceptible to cracking. The crack size of the merged molten pool, located at the edge of the snake-scanning-fabricated sample, was smaller than that of the OZ of the samples fabricated by stripe partition and chessboard partition scanning. The merged molten pool was formed through the fusion of two molten pools caused by the snake laser scanning path. The powder layers were firstly scanned to and fro by a laser. The merged molten pools were then formed by combination of two adjacent molten pools. This led to the superposition of residual stresses, which are prone to deformation and cracking, and the size, number, and density of cracks increased. The merged molten pool of the sample fabricated by snake scanning was located at the edge, there was no HAZ of the adjacent OZ, and the cracks were small in size and number, and lower in density. For the samples fabricated using the stripe partition or chessboard partition scanning strategies, the superimposed HAZ of the adjacent OZ promoted the growth of the residual stress. After that, new cracks initiated and propagated in the merged molten pool area. As a result, the number, size, and density of cracks in the OZ increased.

**Figure 5:**
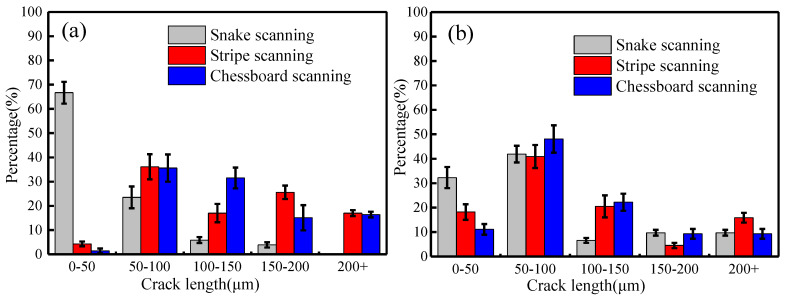

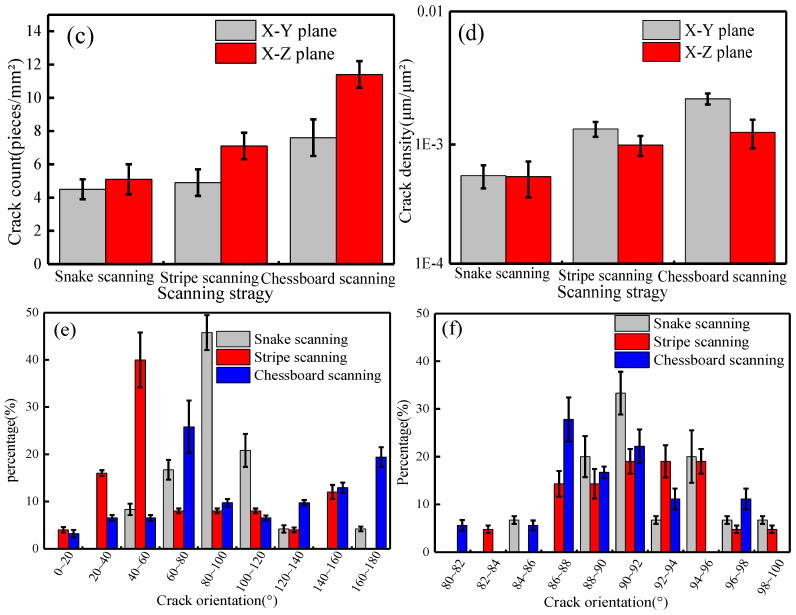
Statistical analysis of cracks in SLMed René104 superalloy samples; (**a**) crack length in the X–Y plane; (**b**) crack length in the X–Z plane; (**c**) crack number; (**d**) crack density; (**e**) crack orientation in the X–Y plane; and (**f**) crack orientation in the X–Z plane.

Figure 5 shows the statistical results of the size, number, density, and orientation of cracks in the SLMed samples. Here, crack size refers to the length of a single crack, number and density of cracks are the number of cracks per unit area, and the total length of crack per unit area, respectively; and crack orientation is defined as the angle between the line connecting the two ends of the crack and X-direction.

The crack size significantly differed among the samples fabricated by the three scanning strategies. In the X–Y plane (Figure 5a), the cracks of sample that was fabricated by snake scanning were mainly composed of small cracks (<50 µm)—accounted for 66.7%—and cracks >200 µm were not observed. The large cracks (>50 µm) of the stripe-partition-scanned samples reached 95.7%, wherein the crack ratio of the size of >200 µm was 17.0%. The number of large cracks (>50 µm) in the chessboard-partition-scanned samples further increased, accounting for 98.6%, and the cracks >200 µm accounted for 16.54%. In the X–Z plane (Figure 5b), all three scanning strategies were yielded large cracks (>50 µm), which reached 67.7% for snake scanning, 71.8% for stripe partition scanning, and 88.9% for chessboard partition scanning and penetrated multiple molten pools and build layers.

Figure 5c shows the statistical result of the crack number of as-fabricated samples. In the X–Y and X–Z planes, the crack number per unit area of the snake-scanned sample was small and was 4.5, and 5.1 stripes/mm^2^, respectively. The highest number of cracks was observed in the chessboard-partition-scanned sample, and reached up to 7.6, and 11.4 stripes/mm^2^, respectively. The crack density of stripe-partition-scanned samples was 4.9 and 7.1 stripes/mm^2^.

Figure 5d shows the statistical result of the crack density of the as-fabricated samples. In the X–Y and X–Z planes, the crack density of all the snake-scanned samples was the lowest–5.6 × 10^−4^ and 5.5 × 10^−4^ µm/μm^2^, respectively–while that of the chessboard-partition-scanned samples was the highest, 22.8 × 10^−4^, and 12.5 × 10^−4^ µm/μm^2^, respectively. The crack density of stripe-partition-scanned samples was in the middle and was 13.3 × 10^−4^ and 9.9 × 10^−4^ µm/μm^2^ in the X–Y and X–Z planes, respectively.

Figure 5e shows that the crack orientation in the X–Y plane of the samples, fabricated by the three scanning strategies, was relatively dispersed. The crack orientation of the snake-scanned sample was concentrated at 80°–100°–accounting for 45.8%, while the crack orientation dispersion of the sample fabricated by chessboard partition scanning was the largest–only 25.8% of which was concentrated at 60°–80°. In the X–Z plane (Figure 5f), the crack orientation of the samples fabricated by three scanning strategies was mainly concentrated between 80° and 100° and propagated along the build direction.

The merged molten pool in the X–Y plane of the sample fabricated by snake scanning was located at the edge of the build layer (Figure 4b), with a length of approximately 1100 µm. No merged molten pool was present inside the as-fabricated sample, which decreased the influence on the internal cracking of the as-fabricated sample, with the cracks mainly originating from the cracking of a single molten pool. Therefore, the crack inside the sample, fabricated by snake scanning, had a small size, number, and a low density (Figure 5a–d).

The thickness of the build layer designed in the present work was 30 µm, and the majority of the crack lengths in the X–Z plane (Figure 3b,d,f and Figure 5b) were >50 µm, which was larger than the thickness of the build layer. The build layers were rotated 67° from each other, and the crack on the single molten pool split along the normal direction of the molten pool. The cracks observed in the X–Y plane included cracks in the current build layer and one or more previous layers. Therefore, the crack orientation of the sample fabricated by snake scanning was relatively dispersed.

The stripe partition scanning strategy increased the stripe OZ compared with the snake scanning strategy. The merged molten pools in the OZ were interconnected, and the HAZs interacted with each other, resulting in large cracks that formed in the merged molten pool in the OZ and passed through multiple molten pools and build layers (Figure 3c,d, Figure 4c and Figure 5a,b). Therefore, the cracks observed in the X–Y plane of the sample, fabricated by stripe partition scanning, contained cracks from single molten pools and the merged molten pools in OZs. In particular, the cracks in the merged molten pools of the OZs came from a plurality of OZs between build layers. The crack orientation was more dispersed than that of the sample fabricated by snake scanning, and the size, number, and density of cracks increased.

The grid size of the sample fabricated by chessboard partition scanning was 5 × 5 mm; a total of 25 checkboard grids were present in the X–Y plane and the grids overlapped each other. The number of OZs and merged molten pools were multiplied, compared with those of the samples fabricated by snake scanning and stripe partition scanning. The laser scanning directions of adjacent grids were orthogonal (Figure 1d), and the molten pools of the adjacent grids in the same build layer were perpendicular to each other. Thus, the size, number, and density of the cracks further increased, and the orientation of the cracks was more dispersed (Figure 3e, Figure 4d and Figure 5e). The merged molten pool and OZ of the samples fabricated by partition scanning cracked more easily, thereby, increasing the crack size, number, and density and decreasing the relative density of the as-fabricated samples. Thus, cracking was the main factor that decreased the relative density of the as-fabricated samples. Therefore, it is necessary to reduce or avoid the formation of OZs of partition scanning by designing a scanning strategy.

### 3.2. Residual Stress

**Figure 6:**
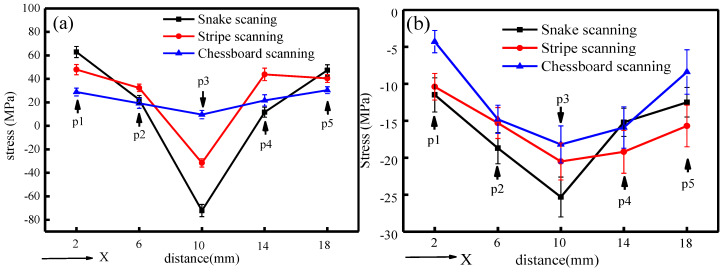
Residual stress distribution of SLMed René 104 superalloy sample along the X-direction in the X–Y plane; (**a**) normal stress; and (**b**) shear stress.

Figure 6 shows the results of the residual stress measured along the X-direction of the X–Y plane of the as-fabricated samples. Figure 6a represents the normal stress distributions, indicating that residual tensile stress was high at the edge and low in the central part of the sample. The residual stress and its distribution in the samples, fabricated using different scanning strategies, were varied. The residual stress at the edge of the snake-scanned samples was a 47.3–62.8 MPa tensile stress, which decreased from the edge to the center when it became the compressive stress, up to 72.3 MPa. In the meantime, stress differences between the edge and central point were calculated as 119.6–135.1 MPa. The increase of the partition numbers reduced the stress differences, which were 72–79.5 and 19.1–20.7 MPa for the stripe partition scanning, and the chessboard partition scanning, respectively. Figure 6b illustrates the distribution of shear stress, as the residual compressive stress. The residual stress differences between the edge and the center of the samples fabricated by the three strategies were gradually decreased with increasing partition numbers. The residual stress distribution in the chessboard-partition-scanned samples became the most uniform.

Residual stress is a critical factor in the cracking behavior of the samples fabricated by SLM [42]. Firstly, different heat dissipation conditions were varied in the X–Y plane of the as-fabricated samples, which led to discrepancies in the residual stress [41,43]. Moreover, during the SLM re-melting process, each new layer results in accumulated residual stresses, which causes cracks to initiate and propagate across the multiple molten pools and build layers. The stripe partition and chessboard partition scanning strategies reduced the laser track length, compared with the snake scanning strategy, which strongly influenced the thermal history during the SLM process. Meanwhile, the partition scanning strategy has a pre-heating effect on adjacent zones, thereby dropping the thermal gradient. Hence, the temperature field of the adjoining scanning zone became uniform, and the corresponding residual stress was also reduced [18,21,22,44]. In this work, the grid size of the sample fabricated using the chessboard partition scanning strategy was the smallest and obtained the lowest thermal gradient and uniform temperature field. Therefore, these samples acquired the lowest and the most balanced residual stress, as the measured value illustrated in this work, which is consistent with the results of Inconel 718 [21] and 316L stainless steel [18,44]. However, the cracking behavior was the opposite (Figure 3 and Figure 5). The samples fabricated by snake scanning had the highest residual stress with some short cracks. Although the residual stress of the chessboard-partition-scanned samples was the lowest, it illustrated many more cracks, which indicates that the partition scanning had no inhibitory effect on the cracking behavior of the SLMed René 104 alloy. Since the number, size, and density of cracks in the chessboard-partition-scanned samples were significantly increased, more residual stress was also released during the propagation of cracks. Thus, the measured residual stress was low and uniformly distributed. It is impractical to quantify the relationship between the cracks density and released stress. Therefore, the actual residual stress in as-SLMed René 104 alloy is difficult to predict. According to the results from this work, it is necessary to design new scanning strategies with optimized scanning partitions and overlaps for acquiring a high-quality, near fully dense, low-weldable nickel-based superalloy using SLM.

## 4. Conclusions

Three scanning strategies of snake, stripe partition and chessboard partition were used to prepare René 104 Ni-based superalloy and proved to have significant effects on the cracks, residual stress, and relative density of the SLMed parts. The major findings can be summarized as follows:

(1) The cracks appeared in all samples, which utilized three varied scanning strategies with different partition numbers. Only a few pore defects were observed in these samples. The partition scanning strategy cannot reduce the cracks in René 104 superalloy. The cracking performance is the most severe in the chessboard-partition-scanned sample, and relatively moderate in the snake-scanned sample.

(2) The cracks were mainly observed in the molten pool of the SLMed René 104 superalloy, which extended along the normal direction of the molten pool. The merged part at the end of adjacent molten pools cracked more significantly compared to that of the middle area, especially in the overlapping zone (OZ) of the neighboring partitions.

(3) The OZ of the scanning partition was sensitive to cracking. The OZ rose with the increase of the numbers of scanning partition zones, resulting in the growth of the number, size and density of cracks. A severe cracking phenomenon appeared in the partition scanned OZ, of which the crack size was up to 460 μm in the stripe-partition-scanned samples, and 700 μm in the chessboard-partition-scanned samples. The snake-scanned samples without OZ only showed cracks with a maximum size of 380 μm.

(4) The cracking results in the relief of residual stresses and the decrease of the relative density of the SLMed René 104 superalloy. Among the three scanning strategies, the snake-scanned samples possess the highest residual stress, the relative density of which was 98.75%, and had the smallest number of cracks. The relative density of the stripe-partition-scanned sample was decreased to 98.17%, and was observed to have more cracks. The residual stress of the chessboard-partition-scanned samples also dropped, and the relative density was reduced to only 98.01%, which can be attributed to the occurrence of the most severe cracking in the three scanning strategies.

According to the above four aspects, it is necessary to design scanning strategies with optimized scanning partitions and overlaps to avoid cracking and acquire the high-quality, near fully dense, low-weldable nickel-based superalloy using SLM.

## Figures and Tables

**Table 1:** Composition of atomized René 104 Ni-based superalloy powder (wt. %).

Element	Co	Cr	Al	Ti	Mo	W	Nb	Ta	Zr	B	C	Ni
Normal	20.6	13	3.4	3.9	3.8	2.1	0.9	2.4	0.05	0.03	0.04	Bal
Measured	20.0	12.6	3.78	2.14	3.24	3.66	2.05	0.82	0.057	0.045	0.05	Bal
